# Usefulness of Modern Activity Trackers for Monitoring Exercise Behavior in Chronic Cardiac Patients: Validation Study

**DOI:** 10.2196/15045

**Published:** 2019-12-19

**Authors:** Cyrille Herkert, Jos Johannes Kraal, Eline Maria Agnes van Loon, Martijn van Hooff, Hareld Marijn Clemens Kemps

**Affiliations:** 1 Máxima Medical Center Flow, Center for Prevention, Telemedicine and Rehabilitation in Chronic Disease Eindhoven Netherlands; 2 Máxima Medical Center Department of Sports Medicine Eindhoven Netherlands

**Keywords:** cardiac diseases, activity trackers, energy metabolism, physical activity, validation studies

## Abstract

**Background:**

Improving physical activity (PA) is a core component of secondary prevention and cardiac (tele)rehabilitation. Commercially available activity trackers are frequently used to monitor and promote PA in cardiac patients. However, studies on the validity of these devices in cardiac patients are scarce. As cardiac patients are being advised and treated based on PA parameters measured by these devices, it is highly important to evaluate the accuracy of these parameters in this specific population.

**Objective:**

The aim of this study was to determine the accuracy and responsiveness of 2 wrist-worn activity trackers, Fitbit Charge 2 (FC2) and Mio Slice (MS), for the assessment of energy expenditure (EE) in cardiac patients.

**Methods:**

EE assessed by the activity trackers was compared with indirect calorimetry (Oxycon Mobile [OM]) during a laboratory activity protocol. Two groups were assessed: patients with stable coronary artery disease (CAD) with preserved left ventricular ejection fraction (LVEF) and patients with heart failure with reduced ejection fraction (HFrEF).

**Results:**

A total of 38 patients were included: 19 with CAD and 19 with HFrEF (LVEF 31.8%, SD 7.6%). The CAD group showed no significant difference in total EE between FC2 and OM (47.5 kcal, SD 112 kcal; *P*=.09), in contrast to a significant difference between MS and OM (88 kcal, SD 108 kcal; *P*=.003). The HFrEF group showed significant differences in EE between FC2 and OM (38 kcal, SD 57 kcal; *P*=.01), as well as between MS and OM (106 kcal, SD 167 kcal; *P*=.02). Agreement of the activity trackers was low in both groups (CAD: intraclass correlation coefficient [ICC] FC2=0.10, ICC MS=0.12; HFrEF: ICC FC2=0.42, ICC MS=0.11). The responsiveness of FC2 was poor, whereas MS was able to detect changes in cycling loads only.

**Conclusions:**

Both activity trackers demonstrated low accuracy in estimating EE in cardiac patients and poor performance to detect within-patient changes in the low-to-moderate exercise intensity domain. Although the use of activity trackers in cardiac patients is promising and could enhance daily exercise behavior, these findings highlight the need for population-specific devices and algorithms.

## Introduction

### Background

Improving physical fitness and physical activity (PA) levels are core components of cardiac rehabilitation (CR) and secondary prevention in patients with coronary artery disease (CAD) and chronic heart failure (CHF) [1,2]. Exercise-based CR in these patients has documented positive effects on psychological well-being, hospitalization, and mortality by slowing the progression of CAD and CHF, while also combatting risk factors as hypertension, dyslipidemia, and psychological stress [1,3,4].

Besides exercise training, enhancing daily PA and reducing sedentary behavior are also effective for the prevention of repetitive cardiac events and therefore highly recommended by the current guidelines [5]. Yet, despite these recommendations, patients with CAD and CHF are characterized by a less active lifestyle compared with individuals not diagnosed with these conditions. Subsequently, this results in further deterioration of their activity level, social participation, prognosis, and quality of life [6-8]. One of the explanations for low activity levels in these patients is that secondary prevention programs and center-based CR typically do not focus on increasing daily activity behavior and reduction of sedentary time, as shown by high sedentary levels in post-CR patients comparable with patients who have not participated in CR [9]. This may be because of the fact that CR programs mainly focus on short-term improvements of exercise capacity rather than preparation for self-management and improving exercise behavior in daily life, causing a relapse into sedentary behavior after the rehabilitation program [10].

Home-based exercise programs such as telerehabilitation, may be successful methods for improving PA and reducing sedentary behavior on long term [11-15]. Telerehabilitation programs do thereby not only focus on prescribing exercise sessions but also on monitoring PA levels during the day to give appropriate feedback on PA behavior. To monitor and promote PA successfully during cardiac telerehabilitation, reliable and nonobtrusive devices to assess energy expenditure (EE) need to be available. In fact, safety and clinical effectivity of medical devices are high on the agenda of the European Society of Cardiology, mainly focusing on high risk (implantable) devices such as coronary artery stents or implantable cardioverter defibrillators [16]. At this stage, less attention is payed to the clinical effectiveness of wearable sensors and its algorithms in cardiac patients. Since nowadays, exercise prescriptions in cardiac patients are being made based on commercially available activity trackers, more information on the validity of these devices in cardiac patients is urgently needed, as medical advice based on invalid physiological parameters might be potentially harmful.

Important prerequisites for activity trackers include high accuracy and responsiveness. Accuracy is defined as the closeness of agreement between the device measurement and the true value [17]. Responsiveness of a device is the ability to detect within-patient changes of exercise intensity over time and is therefore highly important in cardiac patients to monitor progression, and for daily coaching [18,19].

Kraal et al found that combining heart rate (HR) with accelerometer data provides a higher accuracy for measuring EE than using accelerometer or HR data alone in patients with CAD [20]. However, in this study, a hip-worn accelerometer was combined with a chest belt to record HR, which is not feasible for all-day use. Wrist-worn activity trackers can provide a nonobtrusive solution combining HR and accelerometer data for calculation of EE. However, little information is available about their accuracy and responsiveness. Studies that did validate such devices showed mixed results [21-24]. These studies, however, used early device models and were performed in a population without cardiac conditions. The results of these studies cannot be extrapolated to cardiac patients because of factors such as chronotropic incompetence and use of HR lowering medication such as beta blockers. Moreover, to our knowledge, there has been no previous investigation to the responsiveness of wrist-based devices combining HR with accelerometer data, especially not in cardiac patients.

### Objectives

The aim of this study is to investigate the accuracy and responsiveness of 2 commercially available wrist-worn activity trackers to calculate EE in patients with CAD and CHF. This study may provide important information whether 2 modern activity trackers, Fitbit Charge 2 (FC2) and Mio Slice (MS), can be used for measuring EE to monitor PA levels in cardiac patients.

## Methods

### Study Population

Patients (aged ≥18 years) were included based on their diagnosis to form 2 patient groups: patients with stable CAD with preserved left ventricular ejection fraction (LVEF) and patients with stable heart failure with reduced ejection fraction (HFrEF). These 2 patient categories were selected because HFrEF patients generally have lower activity levels compared with CAD patients with preserved LVEF. Household activities, for example, can be experienced as more intensive by HFrEF patients than by CAD patients with preserved LVEF. Also, HFrEF patients often suffer from chronotropic incompetence, yielding a difference in HR variation during activities. Therefore, both groups were analyzed separately. The participants were recruited via their cardiologist in the outpatient clinic of the Máxima Medical Center, the Netherlands, and were randomly selected from a list of patients who participated in previous studies and gave informed consent to be contacted for participation in future research projects. Patients were excluded if they suffered from hemodynamic significant valvular disease, permanent atrial fibrillation or peripheral vascular, neurological, or orthopedic conditions impairing exercise capacity. All patients provided written informed consent. The study was approved by the local medical ethical committee of the Máxima Medical Center and was conducted in accordance with the declaration of Helsinki.

### Protocol

Participants completed a laboratory protocol consisting of 14 low-to-moderate intensity activities, which was modified from a previous study with cardiac patients [20]. The protocol comprised sedentary and household activities, treadmill walking on 3 different speeds, cycling on 3 different loads, and walking up and down the stairs. Walking speeds and cycling loads were adjusted for the HFrEF group. The total duration of the protocol was 39 min (resting time excluded). An overview of the protocol is shown in Table 1. Resting HR was measured using a chest belt (Polar T31, Polar) at the start of the protocol. Between each activity, the patients received recovery time which lasted until the HR reached resting HR. The protocol was performed at the gym of the physical therapy department in the Máxima Medical Center and was supervised by a medical doctor and an assistant. The room temperature was approximately 20°C. EE calculated by the activity trackers was noted at the start and at the end of an activity. To ensure continuous HR tracking during each activity, the workout mode was turned on in both activity trackers when an activity was started.

**Table 1:** Activity protocol.

Activity type and activity		Duration in minutes
**Sedentary activities**		
	Sitting	5
	Standing	2
	Typing	3
**Household activities**		
	Table cleaning	3
	(Un)loading the dishwasher	3
	Vacuuming	3
**Stairs**		
	Ascending	1
	Descending	1
**Cycling (ergometer); load**		
	CAD^a^ 0 Watt; HFrEF^b^ 0 Watt	3
	CAD 40 Watt; HFrEF 25 Watt	3
	CAD 70 Watt; HFrEF 50 Watt	3
**Walking (treadmill); speed/inclination**		
	CAD 4 km/h; HFrEF 3 km/h	3
	CAD 5.5 km/h; HFrEF 4.5 km/h	3
	CAD 4 km/h 5% slope; HFrEF 3 km/h 5% slope	3

^a^CAD: coronary artery disease.

^b^HFrEF: heart failure with reduced ejection fraction.

### Criterion Measure

Breath-by-breath oxygen uptake (VO_2_) and carbon dioxide production (VCO_2_) were measured during the entire length of the protocol using the Oxycon Mobile (OM; CareFusion). The OM is a light-weighted mobile device consisting of a facemask and a gas analyzer unit with battery attached to the patients back via a shoulder belt system. Real time data measured by the device were sent to a computer with corresponding software. Before the start of the protocol, automatic volume and gas calibration was performed and ambient conditions were checked. The OM has been validated before by comparing it with the golden standard, the Douglas Bag, and has been found reliable as a criterion measure [25].

### Devices

#### Fitbit Charge 2

The FC2 (Fitbit Inc) is a wrist-worn activity tracker consisting of a 3-axial accelerometer, an altimeter, and an optical HR tracker. EE calculation is based on a combination of basal metabolic rate (which is calculated by gender, age, height, and weight), activity counts during the activities, and, as claimed by the manufacturer on HR [26,27]. The Fitbit was worn on the dominant wrist following the recommendations of the manufacturer. The activity tracker was connected via Bluetooth to the Fitbit app in which parameters such as date of birth, length, weight, gender, wrist orientation, and handedness were entered for each patient. The app was supplied with the most recent firmware updates.

#### Mio Slice

The MS (MIO Global) is a wrist-worn activity tracker consisting of a 2-axial accelerometer and an optical HR tracker. The activity tracker was worn on the nondominant wrist. Age, length, weight, gender, and wrist orientation were entered in the corresponding app for every patient. Afterwards, the activity tracker was synchronized with the Mio app via Bluetooth. The app contained the latest version of firmware. Information on algorithms used to calculate EE is not provided by the manufacturer. The manufacturer claims that both activity counts and HR are used for EE calculation when the workout mode is activated [28].

### Data Analysis

Raw data from the breathing analysis was exported and imported together with the values from the FC2 and MS in a custom-made MATLAB analysis program (R2018a [9.4.0.813654], Mathworks). The entire activity bouts were analyzed. First, the EE was calculated from breath-by-breath measurements using the Weir equation, as follows [29]:

EE=[(3.941×VO_2_)+(1.11×VCO_2_)]×1.1440

Then, outliers (eg, coughing) in the EE data were detected using a Hampel filter. Outliers were replaced if the value exceeded 3 standard deviations from the median of itself and 3 neighboring data points of that median value [30]. Thereafter, the data were cubic spline interpolated to 1 second values following a 1-Hz low pass fourth Butterworth filter with a cutoff frequency of 0.04 Hz [31,32].

### Statistical Analysis

To achieve 80% power to detect an intraclass correlation coefficient (ICC) of 0.75 (excellent agreement) under the alternative hypothesis that the ICC is 0.35 (poor agreement), a sample size of 19 subjects per study group (ie, CAD and HFrEF) was calculated.

Descriptive statistics were used to describe the population regarding baseline clinical characteristics. Normality of data was assessed by visual inspection of histograms and by interpreting Skewness and Kurtosis [33]. Accuracy of FC2 and MS was assessed by calculating mean EE and mean differences in EE compared with the criterion measure (OM). The values were calculated per activity and over the total protocol (resting time included). To identify if agreement (between the activity trackers and the criterion measure) was between reasonable limits (set at 10% error zone), one-sample T-tests were performed using mean differences (device minus OM) compared with zero. In addition, Bland-Altman plots were created to illustrate the level of agreement between estimated EE and criterion EE with mean bias and 95% upper and lower limits of agreement (LoA). Data falling outside the LoA were inspected; however, no clear reason was found why these data were different. Therefore, these data were included in the analysis. The ICC using 2-way mixed models with absolute agreement was used for assessment of reliability of the devices for each activity and total protocol. An ICC below 0.4 was considered poor, between 0.4 and 0.59 fair, between 0.6 and 0.74 good, and above 0.75 was considered as excellent reliability [34]. The root mean square error (RMSE) of FC2 and MS was calculated for the total protocol in both groups. Responsiveness of FC2 and MS was assessed by using a paired T-test during walking at different speeds and cycling at different loads. All data analyses were performed using SPSS software (Version 22.0, SPSS Inc). Significance level was set at *P*<.05 for all analyses.

## Results

A total of 38 patients were included and completed the protocol. The group was equally divided in CAD patients (n=19, age 61.4 years, SD 6.9 years) and patients with HFrEF (n=19, age 65.1 years, SD 6.6 years, LVEF 31.8%, SD 7.6%). In both groups, the majority of patients were using HR lowering medication (14 CAD patients [14/19, 74%] and 17 HFrEF patients [17/19, 89%]). Pulmonary diseases were present in 2 HFrEF patients (1 patient with mild bronchiectasis and 1 patient with COPD treated by the general practitioner). Additional patient characteristics are shown in Table 2. All data recordings of 2 patients (1 CAD and 1 HFrEF) and stair walking activities of 3 additional (2 CAD and 1 HFrEF) were excluded from the analysis, because of a failure in OM measurement.

**Table 2:** Patient characteristics.

Characteristics		CAD^a^ (N=19)		HFrEF^b^ (N=19)	
Age (years), mean (SD)		61.4 (6.9)		65.1 (6.6)	
**Gender, n (%)**					
	Male		14 (74)		17 (89)
	Female		5 (26)		2 (11)
Height (cm), mean (SD)		176 (6.8)		177 (5.4)	
Weight (kg), mean (SD)		84.3 (12.1)		86.7 (13.7)	
BMI^c^ (kg/m^2^), mean (SD)		27.1 (3.1)		27.7 (4.2)	
LVEF^d^ (%), mean (SD)		60.5 (4.5)		31.8 (7.6)	
NYHA^e^ classification I/II/III/Unknown, n (%)		—^f^		6(32)/11(58)/1(5)/1(5)	
**Heart failure etiology, n (%)**					
	Ischemic		—		11 (58)
	Nonischemic		—		8 (42)
**Medication, n (%)**					
	Beta-blocker		12 (63)		15 (79)
	Calcium channel blocker (non-DHP^g^)		2 (11)		0 (0)
	Amiodarone		0 (0)		4 (21)
	Ivabradine		0 (0)		1 (5)

^a^CAD: coronary artery disease.

^b^HFrEF: heart failure with reduced ejection fraction.

^c^BMI: body mass index.

^d^LVEF: left ventricular ejection fraction.

^e^NYHA: New York Heart Association.

^f^Not applicable.

^g^DHP: dihydropyridine.

### Accuracy

#### Coronary Artery Disease Group

**Figure 1:**
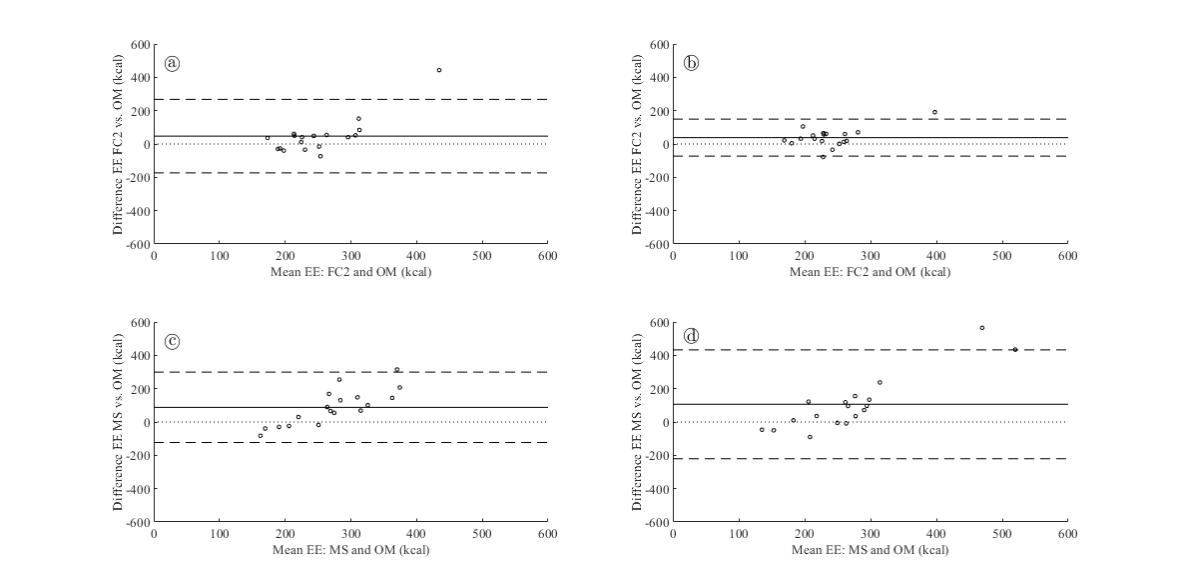
Bland-Altman plots for total energy expenditure. The solid horizontal line corresponds to the mean difference, whereas the dashed horizontal lines correspond to limits of agreement. The dotted line is the line of equality. (a) Comparison of Oxycon Mobile with Fitbit Charge 2 for patients with CAD. (b) Comparison of Oxycon Mobile with Fitbit charge 2 for patients with HFrEF. (c) Comparison of Oxycon Mobile with Mio Slice for patients with CAD. (d) Comparison of Oxycon Mobile with Mio Slice for patients with HFrEF. CAD: coronary artery disease; EE: energy expenditure; FC2: Fitbit Charge 2; HFrEF: heart failure with reduced ejection fraction; MS: Mio Slice; OM: Oxycon Mobile.

The Multimedia Appendix 1 demonstrates the accuracy of EE measurement by FC2 and MS for participants with CAD. Mean (SD) EE in the CAD group over the total protocol was 228.1 (37.0) kcal, 275.6 (113.5) kcal, and 316.2 (113.3) kcal for OM, FC2, and MS, respectively. MS significantly overestimated EE over the total protocol (mean difference 88.1 kcal, *P*=.003). FC2 showed a nonsignificant overestimation in total EE (mean difference 47.5 kcal, *P*=.09). Most sedentary activities were underestimated by both FC2 and MS, and cycling activities were underestimated by FC2 at all loads. Bland-Altman plots based on total EE illustrate the overestimation with wide LoA for both devices (see Figure 1). The MS showed an increasing bias when EE levels are higher. The ICCs for the total protocol were low for both devices (FC2 0.10 and MS 0.12). The RMSE was 119.2 kcal and 136.7 kcal for FC2 and MS, respectively.

#### Heart Failure With Reduced Ejection Fraction Group

The Multimedia Appendix 2 demonstrates the accuracy of EE measurement by FC2 and MS for participants with HFrEF. Mean (SD) EE over the total protocol in the HFrEF group was 218.2 (42.3) kcal, 256.4 (69.3) kcal, and 324.4 (174.6) kcal for OM, FC2, and MS, respectively. Both devices significantly overestimated EE (mean difference FC2 38.2 kcal, MS 106.2 kcal, *P*=.01 and *P*=.02, respectively) with a similar pattern of underestimation in sedentary activities and cycling activities. Bland-Altman plots based on total EE illustrate the overestimation with wide LoA for both devices (see Figure 1). The MS showed wider LoA (lower LoA −220.3 kcal and upper LoA 432.7 kcal) and an increasing bias when EE levels were higher. The ICCs for the total protocol were low for both devices (FC2 0.42 and MS 0.11). The RMSE was 66.9 kcal and 193.6 kcal for FC2 and MS, respectively.

### Responsiveness

Table 3 shows the ability of FC2 and MS to detect within patient changes in walking and cycling activities.

#### Coronary Artery Disease Group

FC2 was able to detect a difference between cycling at 0 versus 40 watts (mean difference 3.3 kcal, *P*=.003) and between walking at 4 km/h with a 5% slope versus 5.5 km/h (mean difference 4.4 kcal, *P*=.01) in patients with CAD. However, no significant differences were observed for the other walking and cycling activities. The MS was able to detect differences at all cycling loads; however, it was not able to detect any differences in walking speeds/inclination. Note that the difference in EE between walking 4 km/h with a 5% slope and 5.5 km/h was nonsignificant as measured by the OM.

#### Heart Failure With Reduced Ejection Fraction Group

FC2 was not able to detect changes at any walking speeds or cycling loads in the HFrEF group. MS was able to detect within-patient changes at cycling 0 versus 50 watts (mean difference 4.7 kcal, *P*=.02), at cycling 25 versus 50 watts (mean difference 3.6 kcal, *P*=.02) and at walking 3 km/h with a 5% slope versus 4.5 km/h (mean difference 3.0 kcal, *P*=.03). Note that the difference in EE between walking 3 km/h with a 5% slope and 4.5 km/h was nonsignificant as measured by the OM.

**Table 3:** Responsiveness of Fitbit Charge 2 and Mio Slice.

Group and activity			Oxycon Mobile, Mean difference (kcal^a^)	*P* value	Fitbit Charge 2, Mean difference (kcal)	*P* value	Mio Slice, Mean difference (kcal)	*P* value
**CAD^b^** **(N=18)**								
	**Cycling**							
		0 versus 40 watts	2.7	<.001	3.3	.003	3.2	<.001
		0 versus 70 watts	5.4	<.001	2.6	.11	5.1	<.001
		40 versus 70 watts	2.7	<.001	0.7	.67	1.9	.03
	**Walking**							
		4 km/h versus 4 km/h 5% slope	2.5	<.001	1.8	.15	2.2	.36
		4 km/h versus 5.5 km/h	2.5	<.001	2.6	.15	3.8	.31
		4 km/h 5% slope versus 5.5 km/h	0.1	.71	4.4	.01	1.6	.42
**HFrEF^c^** **(N=18)**								
	**Cycling**							
		0 versus 25 watts	1.2	<.001	0.3	.88	1.1	.23
		0 versus 50 watts	3.0	<.001	1.0	.46	4.7	.02
		25 versus 50 watts	1.9	<.001	1.3	.40	3.6	.02
	**Walking**							
		3 km/h versus 3 km/h 5% slope	1.1	.002	1.9	.16	.8	.66
		3 km/h versus 4.5 km/h	1.5	.001	.3	.89	2.2	.16
		3 km/h 5% slope versus 4.5 km/h	0.4	.27	2.2	.08	3.0	.03

^a^kcal: kilocalories.

^b^CAD: coronary artery disease.

^c^HFrEF: heart failure with reduced ejection fraction.

## Discussion

This study is the first to evaluate the accuracy and responsiveness of wrist-worn activity trackers combining HR monitoring and accelerometry for EE calculation in patients with CAD with preserved LVEF and patients with HFrEF. Poor accuracy was observed for both devices in predicting EE, with MS performing worse than FC2. MS provides a higher responsiveness than FC2 with regard to the ability to detect changes in cycling load, but both devices performed poorly with respect to detecting within-patient differences in walking speed.

### Accuracy

Both FC2 and MS significantly overestimated EE over the total activity protocol with a tendency of greater bias when EE increased. Other studies using wrist-worn Fitbit models that combine accelerometer data and heart data showed mixed results. The results from our study were in line with previous research from Bai et al which showed a whole-trial overestimation of EE (mean absolute percentage error 32.9%) of FC HR [23]. Regarding exercise intensity, the device showed an underestimation of sedentary activities and overestimation of light physical activities and aerobic activities, similar to our results. Dooley et al also found significant overestimation of EE at baseline, light, and moderate intensity treadmill activities by FC HR [35]. In contrast, other studies showed an underestimation of EE by wrist-worn devices [21,22]. Differences in study outcomes might be due to variation in study design and population. The above-mentioned studies have been performed in noncardiac participants, so results cannot be extrapolated to cardiac patients using beta-blocker medication and chronotropic incompetence. To our knowledge, only 1 study evaluated the accuracy of a wrist-worn Fitbit device (Fitbit Flex, 2013) in a population in which 59% had coronary heart disease [36]. This study showed a 10% overestimation of minutes of moderate to vigorous physical activity (MVPA), which was in line with our study. Although they found a high correlation between Fitbit and the criterion measure for minutes of MVPA (r=0.74 total population, r=0.71 cardiac patients), ICC’s and Bland-Altman analysis were not performed to evaluate accuracy.

Possible causes for the limited accuracy of EE estimation by different wrist-worn devices include poor quality of HR and accelerometer assessment and inadequate algorithms to calculate EE (ie, not well tailored to the target population). Yet, the algorithms to calculate EE are usually not provided by the manufacturer. As patient characteristics such as length, weight, and exercise modality are fixed, EE estimates are most likely determined by the accuracy of the (accelerometer and HR) sensors and the reliability of the algorithms related to HR and activity counts. However, both the reliability of the algorithms and the accuracy of both sensors were not evaluated in this study. Concerning the accuracy of the HR sensor, Wallen et al concluded that Mio Alfa and FC HR slightly underestimate HR, within an expectable range (ICC Fitbit 0.78; ICC Mio 0.91) [21]. Other studies found similar results for Fitbit Surge, FC HR, and Mio Alfa [24,37]. In total, 2 studies found that the error of Mio Alfa was comparable with the reference method; however, considerable variability was observed when dividing protocol activities into different types, and the device did not perform well as speed or load of an activity increased [38,39]. Despite reasonable accuracy of HR assessment by previous Fitbit and Mio models, it remains unclear how HR is incorporated in EE equations. Moreover, it is questionable if the relation between changes in HR and EE is comparable between people who do not have a cardiac condition and patients with CAD or HFrEF. Therefore, EE estimates might be even more inaccurate in our study compared with these studies with individuals without cardiac conditions.

Another factor that may have influenced the accuracy of EE estimation is the location of the accelerometer. Waist placement is generally considered favorable as the sensor is close to the center of body mass and is able to detect whole body movement. A recent review evaluating the influence of body placement to accuracy of EE estimation concluded that wrist placement generally leads to overestimation and torso placement to underestimation of EE, with a greater mean error for devices placed at the wrist [40]. Hand placement on the bars of the treadmill might also contribute to the devices not being able to provide an accurate EE measurement in our study. Wrist-based wearable devices might have difficulties with detecting activities when specific characteristics of an activity (swing of the arm during walking) are not detected. Nonetheless, our findings for indoor conditions are valuable as patients will also use the treadmill during rehabilitation, leisure time sports activities, or even at home.

Nevertheless, our study clearly showed that EE estimates, using algorithms for commercially available wrist-worn devices, should be interpreted with caution in cardiac patients. Therefore, to improve the utility of these devices for this population, extraction of raw HR and accelerometer data are needed to be able to develop adequately tailored algorithms. However, most manufacturers of activity trackers do not provide this opportunity.

### Responsiveness

Whereas, MS was shown to be useful for detecting changes in cycling load, changes in walking speed and inclination were not detected. Furthermore, FC2 was not capable in detecting changes in both walking and cycling activities. Although the ability to detect changes in intensity within specific activities is an important feature of an activity tracker, previous research on responsiveness is scarce. Price et al concluded the hip-worn Fitbit One is able to detect gross changes in walking and running speed [41]. Compared with Price et al [41], we tested responsiveness for smaller differences in walking speed, which may explain the difference with our findings. In addition, Gusmer et al showed the hip-worn Fitbit Ultra was able to detect changes in slow and brisk walking, where slow walking was defined as minus 10% of a self-selected comfortable speed and brisk walking as plus 10% of the self-selected speed [42]. Personalized changes in gait speed and device placement might contribute to the detection of differences in walking speed. To our knowledge, there are no studies available which evaluate responsiveness during cycling, which is relevant because cycling is a major component of Dutch CR and during daily life activities [43]. Given the fact that daily exercise behavior consists of a combination of walking, cycling, and other physical activities, our results show that EE measured by both devices should not be used for monitoring purposes and exercise prescription in patients with CHF and CAD. However, wearable devices have demonstrated promising results in motivating and engaging PA and exercise behavior [44].

### Strengths and Limitations

This study is the first to evaluate both the accuracy and responsiveness of wrist-worn activity trackers, which combine HR and accelerometer data in a cardiac population. The responsiveness of a device is a very important feature when implemented in practice, such as in cardiac telerehabilitation. The study is limited by not evaluating test-retest reliability. This would have given a more complete overview of the overall device validity. Moreover, patients were tested in a laboratory setting, so it is not sure whether these results can be extrapolated to free-living conditions. However, because we mimicked free-living conditions by creating a protocol consisting of daily life activities, we expect little differences with a free-living validation study. Another limitation is that HR boundaries were not personalized for each patient. As we did not assess the maximum HR for each individual patient, default settings of the activity trackers were used, which could have influenced the calculation of EE.

### Conclusions

Both wrist-worn activity trackers demonstrated low accuracy in estimation of EE in patients with CAD and HFrEF. Importantly, both devices also showed poor performance to detect within-patient changes in the low-to-moderate exercise intensity domain. Notwithstanding the fact that the use of activity trackers in cardiac patients might stimulate daily exercise behavior, these findings highlight the need for population-specific devices and algorithms.

## Appendix

Multimedia Appendix 1 Accuracy of energy expenditure measurement by Fitbit Charge 2 and Mio Slice, for participants with coronary artery disease.

Multimedia Appendix 2 Accuracy of energy expenditure measurement by Fitbit Charge 2 and Mio Slice, for participants with heart failure with reduced ejection fraction.

## Abbreviations

CAD: coronary artery disease
CHF: chronic heart failure
CR: cardiac rehabilitation
EE: energy expenditure
FC2: Fitbit Charge 2
HFrEF: heart failure with reduced ejection fraction
HR: heart rate
ICC: intraclass correlation coefficient
LoA: limits of agreement
LVEF: left ventricular ejection fraction
MS: Mio Slice
OM: Oxycon Mobile
PA: physical activity
RMSE: root mean square error
