# Supplementary material for: Usefulness of Modern Activity Trackers for Monitoring Exercise Behavior in Chronic Cardiac Patients: Validation Study
Source: JMIR Mhealth Uhealth. 2019 Dec 19;7(12):e15045. doi: 10.2196/15045 (PMC6940867; doi:10.2196/15045)
Supplement: Multimedia Appendix 1 [file mhealth_v7i12e15045_app1.pdf]

Accuracy of energy expenditure measurement by Fitbit Charge 2 and Mio Slice, for participants with CAD[a]

	Heart rate (Polar belt)	Oxycon Mobile (Criterion measure)	Fitbit Charge 2	Mio Slice	Oxycon Mobile vs Fitbit Charge 2				Oxycon Mobile vs Mio Slice			
	**Mean ± SD[b] (beats/min)**	**Mean ± SD[b] (kcal[c])**	**Mean ± SD[b] (kcal[c])**	**Mean ± SD[b] (kcal[c])**	**Mean difference (kcal[c])**	**Lower LoA[d] (kcal[c])**	**Upper LoA[d] (kcal[c])**	**ICC[e]**	**Mean difference (kcal[c])**	**Lower LoA[d] (kcal[c])**	**Upper LoA[d] (kcal[c])**	**ICC[e]**
Sedentary activities												
• *Standing*	72 ± 12	2.8 ± 0.5	2.7 ± 1.5	0.7 ± 2.3	-0.1	-3.1	2.8	0.075	-2.1*	-6.8	2.6	0.002
• *Sitting*	66 ± 8	7.0 ± 1.0	6.4 ± 1.1	0.5 ± 0.6	-0.5*	-2.1	1.0	0.663	-6.5**	-8.9	-4.0	-0.002
• *Typing*	73 ± 12	5.1 ± 0.7	4.4 ± 0.7	4.8 ± 6.9	-0.7**	-2.1	0.7	0.310	-0.2	-13.6	13.1	0.041
Household activities												
• *Table cleaning*	83 ± 15	8.8 ± 1.7	13.5 ± 5.2	23.3 ± 6.7	4.7**	-3.4	12.8	0.249	14.5**	3.2	25.9	0.055
• *Dishwasher*	79 ± 14	6.7 ± 1.4	15.2 ± 4.7	20.7 ± 8.0	8.5**	0.9	16.0	0.098	14.0**	-1.7	29.6	0.011
• *Vacuuming*	83 ± 14	8.6 ± 2.1	16.3 ± 5.3	21.3 ± 7.0	7.7**	-1.9	17.3	0.087	12.7**	-0.3	25.7	0.046
Stairs												
• *Ascending*	96 ± 20	4.5 ± 0.9	10.9 ± 4.8	7.7 ± 4.0	6.5**	-2.9	15.8	0.011	3.2*	-3.9	10.4	0.134
• *Descending*	85 ± 18	3.4 ± 0.9	6.8 ± 3.9	9.0 ± 4.9	3.4*	-4.1	11.0	0.021	5.6**	-3.1	14.4	0.086
Walking												
• *4 km/h*	85 ± 12	11.3 ± 1.7	16.9 ± 6.6	22.0 ± 10.0	5.6*	-5.8	17.1	0.157	10.7**	-8.5	29.9	0.037
• *4 km/h 5% incline*	92 ± 14	13.8 ± 1.9	15.1 ± 6.2	24.2 ± 8.1	1.3.	-10.5	13.1	0.131	10.5**	-3.5	24.4	0.108
• *5.5 km/h*	90 ± 22	13.8 ± 2.0	19.4 ± 7.6	25.8 ± 10.5	5.6*	-8.4	19.6	0.117	12.0**	-8.0	32.1	0.038
Cycling												
• *0 W*	80 ± 13	8.0 ± 1.3	6.3 ± 2.8	18.5 ± 7.4	-1.8*	-8.0	4.5	-0.049	10.5**	-2.6	23.6	0.070
• *40 W*	86 ± 12	10.7 ± 1.4	9.6 ± 4.2	21.7 ± 6.9	-1.1	-9.4	7.2	0.083	11.0**	-1.7	23.7	0.040
• *70 W*	94 ± 14	13.4 ± 1.3	8.9 ± 6.0	23.6 ± 6.4	-4.5*	-15.6	6.5	0.109	10.2**	-1.6	21.9	0.051
Total protocol (resting time included)	81 ± 16	228.1 ± 37.0	275.6 ± 113.5	316.2 ± 113.3	47.5	-172.9	267.9	0.101	88.1*	-122.9	299.0	0.123

Mean differences are calculated as device minus criterion measure.

[a]CAD: coronary artery disease

[b]SD: standard deviation

[c]Kcal: kilocalories

[d]LoA: limit of agreement

[e]ICC: intraclass correlation coefficient

*P<0.05, **P<0.001
