# Supplementary material for: Usefulness of Modern Activity Trackers for Monitoring Exercise Behavior in Chronic Cardiac Patients: Validation Study
Source: JMIR Mhealth Uhealth. 2019 Dec 19;7(12):e15045. doi: 10.2196/15045 (PMC6940867; doi:10.2196/15045)
Supplement: Multimedia Appendix 2 [file mhealth_v7i12e15045_app2.pdf]

Accuracy of energy expenditure measurement by Fitbit Charge 2 and Mio Slice, for participants with HFrEF[a]

	Heart rate (Polar belt)	Oxycon Mobile (Reference method)	Fitbit Charge 2	Mio Slice	Oxycon Mobile vs Fitbit Charge 2				Oxycon Mobile vs Mio Slice			
	**Mean ± SD[b] (beats/min)**	**Mean ± SD[b] (kcal[c])**	**Mean ± SD[b] (kcal[c])**	**Mean ± SD[b] (kcal[c])**	**Mean difference (kcal[c])**	**Lower LoA[d] (kcal[c])**	**Upper LoA[d] (kcal[c])**	**ICC[e]**	**Mean difference (kcal[c])**	**Lower LoA[d] (kcal[c])**	**Upper LoA[d] (kcal[c])**	**ICC[e]**
Sedentary activities												
• *Standing*	72 ± 15	3.1 ± 0.8	2.9 ± 0.9	1.4 ± 4.8	-0.2	-1.5	1,0	0.673	-1.7	-11.6	8.2	-0.079
• *Sitting*	70 ± 15	7.4 ± 1.4	7.2 ± 1.4	3.1 ± 12.0	-0.2	-2.8	2.4	0.528	-4.3	-28.3	19.7	-0.034
• *Typing*	73 ± 14	5.3 ± 1.2	5.2 ± 2.1	3.7 ± 8.6	-0.1	-3.2	3.0	0.569	-1.6	-19.0	15.8	-0.046
Household activities												
• *Table cleaning*	80 ± 15	8.4 ± 1.7	12.7 ± 5.5	24.7 ± 9.8..	4.2*	-6.1	14.6	0.101	16.3 **	-1.9	34.4	0.036
• *Dishwasher*	79 ± 15	6.9 ± 1.3	15.0 ± 4.6	22.6 ± 8.7..	8.1**	-0.7	16.9	0.035	15.7**	-1.5	32.9	0.002
• *Vacuuming*	81 ± 16	9.1 ± 2.3	18.7 ± 4.5	21.6 ± 9.2..	9.7**	1.0	18.3	0.051	12.5**	-5.3	30.4	0.028
Stairs												
• *Ascending*	90 ± 17	3.5 ± 1.2	8.6 ± 4.0	9.5 ± 6.3.	5.1**	-2.9	13.1	0.018	6.1**	-5.2	17.3	0.104
• *Descending*	85 ± 17	3.0 ± 0.7	5.7 ± 2.3	10.8 ± 4.0..	2.7**	-1.4	6.7	0.132	7.8**	0.2	15.4	0.018
Walking												
• *3 km/h*	86 ± 18	10.9 ± 2.2	17.8 ± 6.5	22.7 ± 11.8.	7.0**	-4.1	18.1	0.166	11.8**	-10.2	33.8	0.069
• *3 km/h 5% incline*	88 ± 18	11.9 ± 2.8	15.9 ± 6.0	21.9 ± 8.9..	3.9*	-7.9	15.8	0.129	9.9**	-5.5	25.4	0.134
• *4.5 km/h*	91 ± 19	12.3 ± 2.8	18.1 ± 6.8	24.9 ± 9.2..	5.8*	-9.0	20.6	-0.035	12.6**	-3.6	28.8	0.096
Cycling												
• *0 W*	80 ± 16	8.2 ± 1.4	8.9 ± 6.0	18.8 ± 9.3..	0.7	-10.1	11.5	0.215	10.6**	-6.9	28.0	0.050
• *25 W*	83 ± 16	9.4 ± 1.5	9.2 ± 6.3	19.9 ± 8.6..	-0.2	-11.5	11.2	0.196	10.5**	-5.5	26.6	0.049
• *50 W*	87 ± 16	11.2 ± 1.6	7.9 ± 4.1	23.5 ± 9.4..	-3.3*	-11.3	4.7	0.079	12.3**	-7.0	31.5	-0.023
Total protocol (resting time included)	81 ± 18	218.2 ± 42.3	256.4 ± 69.3	324.4 ± 174.6	38.2*	-72.6	148.9	0.423	106.2*	-220.3	432.7	0.108

Mean differences are calculated as device minus criterion measure
[a]HFrEF: heart failure with reduced ejection fraction
[b]SD: standard deviation
[c]Kcal: kilocalories
[d]LoA: limit of agreement
[e]ICC: intraclass correlation coefficient
*P<0.05, **P<0.001
